# Transgenic Petunia with the Iron(III)-Phytosiderophore Transporter Gene Acquires Tolerance to Iron Deficiency in Alkaline Environments

**DOI:** 10.1371/journal.pone.0120227

**Published:** 2015-03-17

**Authors:** Yoshiko Murata, Yoshiyuki Itoh, Takashi Iwashita, Kosuke Namba

**Affiliations:** Suntory Foundation for Life Sciences, Bioorganic Research Institute, Osaka, Japan; Purdue University, UNITED STATES

## Abstract

Iron is an essential nutrient for all plants. However, terrestrial plants often suffer from iron deficiency in alkaline soil due to its extremely low solubility. Alkaline soil accounts for about 30% of all cultivated ground in the world. Plants have evolved two distinct strategies, I and II, for iron uptake from the soil. Dicots and non-graminaceous monocots use Strategy I, which is primarily based on the reduction of iron(III) to iron(II) and the uptake of iron(II) by the iron-regulated transporter, IRT1. In contrast, graminaceous plants use Strategy II to efficiently acquire insoluble iron(III). Strategy II comprises the synthesis and secretion of iron-chelating phytosiderophores, such as mugineic acids and the Yellow Stripe 1 transporter proteins of the iron(III)-phytosiderophore complex. Barley, which exhibits the highest tolerance to iron deficiency in alkaline soil among graminaceous plants, utilizes mugineic acids and the specific iron(III)-mugineic acids transporter, HvYS1. In this study, we established the transgenic plant *Petunia hybrida*, which originally had only Strategy I, by introducing the *HvYS1* transporter gene derived from barley. When the transgenic plants were grown hydroponically in media containing the iron(III)-2′-deoxymugineic acid complex, free 2′-deoxymugineic acid and its iron(III) complex were detected in the root extract of the transgenic plant by electrospray ionization-Fourier transform-ion cyclotron resonance mass spectrometry. The growth of the transgenic petunia was significantly better than that of the control host in alkaline conditions. Consequently, the transgenic plant acquired a significantly enhanced tolerance to alkaline hydroponic media in the presence of the iron(III)-2′-deoxymugineic acid complex. Furthermore, the flower color of the transgenic plant deepened. The results showed that iron-phytosiderophore complexes and their transporters can potentially be utilized to overcome the worldwide iron uptake problems to diverse plant species that are found in areas with alkaline conditions.

## Introduction

For all plants, iron is an essential element for photosynthesis, DNA synthesis, and many other cellular functions. Because animals ultimately depend on plants for their iron supply, the primary uptake of iron from the soil by plants is very important for all living organisms [1, 2]. Iron is the fourth most abundant constituent of soils, comprising about 5 weight% of the soil [3]. However, plants often suffer from iron deficiency due to the extremely low solubility of iron in alkaline soil, which covers about one-third of the cultivable land on the earth. The concentrations of solubilized iron in the soil is calculated to be only 10^–17^ and 10^–20^ M at pH 7.0 and 8.0, respectively, whereas plants typically require 10^–4^ to 10^–8^ M of iron for their optimal growth [3, 4].

**Fig 1:**
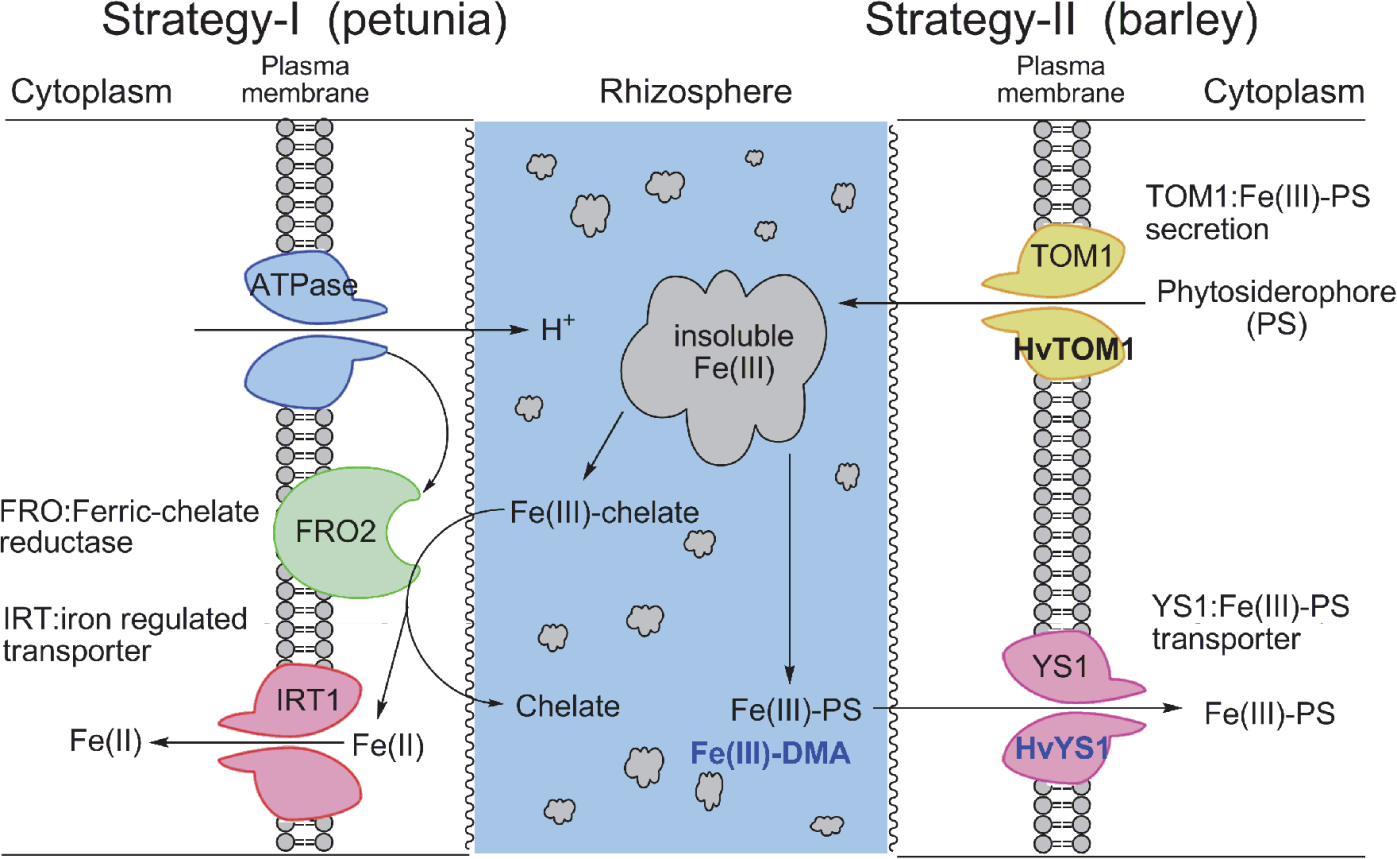
Two distinct strategies for iron uptake in plants. Most plants, including dicots and non-graminaceous monocots, adopt Strategy I, while graminaceous plants utilize Strategy II. In this study, a petunia (Strategy I) was transformed with the *HvYS1* transporter gene *(DDBJ Accession No. AB214183) [23]* for the ion-phytosiderophore complex [Fe(III)-PS] from barley (Strategy II).

Under aerobic conditions, iron takes insoluble forms, such as ferric oxide, Fe_2_O_3_ [5]. Plants have two distinct strategies for iron uptake from the roots (Fig. 1) [6]. Dicotyledonous plants use Strategy I to transport ferrous iron, Fe(II), from soil into the root cells through the iron-regulated transporter 1 (IRT1) after reduction from ferric iron, Fe(III), to Fe(II) by the ferric-chelate reductase FRO2 near the plasma membrane [7–9]. In contrast, graminaceous plants have a unique iron uptake system [10, 11], called Strategy II which is characterized by the synthesis [3, 12–15] of iron-chelating substances, phytosiderophores (PS), which are called mugineic acids (MAs) and which have molecular weights of about 300 and contain six functional groups for iron chelation [16, 17]. MAs are secreted through MAs transporter the TOM1 [18] and absorbed by a specific uptake system as the Fe(III)-MAs complex. The *yellow stripe 1* (*YS1*) gene, which is responsible for the efficient uptake of the Fe(III)-PS complex, was first identified in maize (*Zea mays*) [19]. Further investigations have demonstrated that ZmYS1 is a proton/Fe(III)-PS cotransporter [20] that belongs to the oligopeptide transporter family, which is in bacteria, Archaea, fungi, and plants [21]. The heterologous expression of *ZmYS1* in yeast and *Xenopus* oocytes has shown that ZmYS1 transports PS-bound metals, such as zinc, copper, and nickel [20, 22]. It has also revealed that ZmYS1 transports nicotianamine (NA) in complex with nickel, Fe(II), or Fe(III) [20]. NA is a MA precursor and more importantly acts as an iron transport in a plant.

In barley (*Hordeum vulgare* L.), we have identified a transporter, HvYS1 [23], with high homology to ZmYS1 (72.7% identity and 95.0% similarity). In particular, all predicted transmembrane regions of the two proteins are virtually identical. The expression pattern of the *HvYS1* gene in barley [23] has revealed that these proteins are mostly located in the roots. Furthermore, their expression is enhanced 50-fold under Fe-deficient conditions compared to Fe-sufficient environments. These results suggest that HvYS1 is the transporter for the primary uptake of iron in the soil by barley roots. YS-like proteins (YSL) have been identified in barley. However, they promote the uptake of different substrates and show different localizations from those of HvYS1 [24, 25]. For example, HvYSL2 is localized to the endodermis in the roots, and it transports PS complexed with Fe(III), Zn(II), Ni(II), Cu(II), Mn(II), or Co(II) [24]. HvYSL5 is localized in the vesicles in the roots [25]. In rice (*Oryza sativa* L.), 18 YSL genes have been identified [26]. Among these, OsYSL2 transports Fe(II)-NA and Mn(II)-NA [26], and OsYSL15, OsYSL16, and OsYSL18 transport Fe(III)-2′-deoxymugineic acid (DMA) [27–29]. Another class of YSL proteins in non-graminaceous plants, such as *Arabidopsis*, synthesizes NA but not MAs, and these YSL proteins carry metal ions in plants by interacting with NA [30]. Recently, in *Arabidopsis*, YSL4 and YSL6 have been shown to control iron release from the chloroplast [31].

In this report, we focused on *Petunia hybrida*, which is a plant that use Strategy I, and established transformants by introducing the *HvYS1* transporter gene and the PS, DMA, by which the plant acquired tolerance to iron deficiencies under alkali conditions. DMA is a PS of corn and rice, and we have established a highly efficient synthetic route for supplying a sufficient amount of DMA [32]. We successfully detected both the Fe(III)-DMA complex and free DMA in extracts from the plant roots of transformants by electrospray ionization-Fourier transform-ion cyclotron resonance mass spectrometry (ESI-FT-ICR MS).

## Materials and Methods

### Construction of the HvYS1 expression vector

A full-length cDNA of *HvYS1 (DNA Data Bank of Japan (DDBJ) under the accession number AB214183)* was amplified with the forward 5′-GCTCTAGAATGGACATCGTCGCC-3′ and the reverse 5′-CCCAAGCTTTTAGGCAGCAGGTAG-3′ primers, which were subcloned to the PERII-TOPO vector with a TOPO-TA cloning kit (Life Technologies Corporation, Grand Island, NY, USA). The *HvYS1* cDNA was inserted into the Mac-1 promoter [33] and the mannopine synthase (mas) terminator [34] in the sense orientation. The constructed expression cassette was inserted into a binary vector, pBinPLUS [35], to produce the plasmid Mac-*HvYS1*-mas-pBinPlus shown in supporting information (S1 Fig.).

### Transformation of the petunias

Subsequently, *Agrobacterium tumefaciens* (strain Agl0) [36] was transformed with Mac-*HvYS1*-mas-pBinPlus based on a previously reported method [37]. Then, the transformed agrobacterium was infected to the petunia [*Petunia hybrid* cultivar Safinia Purple Mini (Suntory Flowers Ltd., Osaka, Japan)] to introduce the *HvYS1* translation-region gene into the petunia. All of the plants were kept at 23 ± 2°C with irradiation (60 μE, cold-white fluorescence lamp) for 16 h. When the roots grew to a length of 2 to 3 cm, the transgenic petunia plants were planted in Debco 5140/2 potting mix (sterilized with an autoclave) in a 15-cm cultivation pot. Four weeks later, the plant was replanted into a 15-cm pot with the same potting mix and kept at 23°C with irradiation for 14 h (300 μE, halogenated mercury lamp). The leaves of the transgenic petunia were mashed, and the total RNA was extracted with an RNeasy Plant Mini Kit (QIAGEN GmbH, Hilden, Germany). cDNA was prepared from 1 μg of extracted RNA with a First Strand cDNA Synthesis Kit with the SuperScript^TM^ II RT enzyme (Life Technologies Corporation).

### Expression and localization of HvYS1 in the transgenic petunia

To confirm the presence of *HvYS1* in the transgenic plants, cDNA that was prepared from total RNA that was extracted from the transgenic petunia was used as a template, and it was amplified by polymerase chain reaction (PCR) with the forward primer, 5′-CAATGGTTCTACACTGGAGGCG-3′, and the reverse primer, 5′-CATCAAATCGGCAGAGATAAGCAC-3′. The glyceraldehyde-3-phosphate dehydrogenase (GAPDH) gene was used as a control gene, and the forward primer, 5′-GGTCGTTTGGTTGCAAGAGT-3′, and the reverse primer, 5′-CTGGTTATTCCATTACAACTAC-3′, [38] were used. The PCR product was detected by 1.2 w/v% agarose gel electrophoresis.

For the immunohistochemistry of the transgenic petunia, roots from both transgenic and nontransgenic petunias were fixed with 4% paraformaldehyde, embedded in paraffin, and cut to prepare 5-μm paraffin cross-sections [39]. After these sections were subjected to deparaffinization and blocking with 1% bovine serum albumin in phosphate-buffered saline (PBS) for 1 h at room temperature, HvYS1 immunostaining was performed with a 1:100-purified rabbit synthesis anti-HvYS1 polyclonal antibody [23] for 15 h at 4°C. After washing four times with PBST (PBS + 0.05% Tween 20) for 10 min, these sections were treated for 1 h at room temperature with the secondary antibody Alexa-Fluor 488 (goat anti-rabbit, Life Technologies Corporation) at a 1:1,000 dilution in PBS. After washing four times with PBST, these samples were mounted with Crystal Mount^TM^ (Biomeda Corporation, Foster City, CA, USA). The microscopic analysis was performed with an Eclipse E400 biological microscope (Nikon Corporation, Tokyo, Japan) and AQUA-Lite (Hamamatsu Photonics K.K., Hamamatsu, Japan).

### Growth assay of the transgenic petunia in hydroponic culture

The transgenic and nontransgenic petunias were grown under the same conditions as described above. After acclimation, the plants were grown in hydroponic culture in a greenhouse with modified MGRL media [40] containing 1.75 mM sodium phosphate buffer (pH 5.8 or 8.0), 1.5 mM MgSO_4_, 2.0 mM Ca(NO_3_)_2_, 3.0 mM KNO_3_, 10.3 μM MnSO_4_, 30 μM H_3_BO_4_, 1.0 μM ZnSO4, H_3_BO_4_, 24 nM (NH_4_)_6_Mo_7_O_24_, 130 nM CoCl_2_, 1 μM CuSO_4_, and 20 μM Fe(III)-ethylenediaminetetraacetic acid (EDTA) or Fe(III)-DMA [11]. Fe(III)-DMA was synthesized as previously reported [32]. We monitored the pH of the hydroponic culture medium before replacing the medium every 2 to 3 days. The pH of the medium containing Fe(III)-EDTA, with the initial pH of 5.8 and 8.0, was changed to 5.51–5.64 and 7.35–7.40, respectively. The pH of the medium containing Fe(III)-DMA with the initial pH of 5.8 and 8.0 was changed to 5.65–5.70 and to 7.24–7.32, respectively. After growing for 15 days in the greenhouse, the total length and weight, as well as the lengths and weights of the roots and shoots, of each plant were measured.

### Determination of iron content

Each root was cut and washed for 5 min in a solution containing 5 mM CaSO_4_ and 10 mM EDTA [8]. The roots, shoots, and flowers were dried for one day at 60°C, weighed, digested completely in concentrated HNO_3_ (ca. 14 M) at 110°C, and then dissolved in 1 mL of 2 M HNO_3_. The iron contents were measured with an atomic absorption spectrophotometer (AA-6800, Shimadzu Corporation, Kyoto, Japan).

### MS analysis of the Fe(III)-DMA complex

The root samples that were prepared from nontransgenic and transgenic petunias, which were grown in MGRL medium [40] containing 20 μM Fe(III)-EDTA or Fe(III)-DMA [11] at pH 5.8, were subjected to FT-ICR MS measurements. Each root sample was immediately frozen in liquid nitrogen and homogenized with a four-fold weight of water. The resultant homogenates were centrifuged at 10,000 × *g* for 10 min, and the supernatants were again centrifuged at 100,000 × *g* for 1 h at 4°C. The supernatants thus obtained were applied to gel filtration column chromatography (glass column supernatant: 3 mm × 300 mm; GL Sciences Inc., Torrance, CA, USA) that was packed with Sephadex G-10 on a high-performance liquid chromatography (HPLC) system (Agilent model 1100; UV, 330 nm; water flow, 0.03 mL/min; Agilent Technologies, Santa Clara, CA, USA), and 31–34 fractions were analyzed by nano-ESI-FT-ICR MS with an Apex-Q 94e (Bruker Daltonik GmbH, Bremen, Germany) that was equipped with an ion source of Apollo 2 dual in negative-ion mode. Calibration was performed with NaI (0.1 mg/mL in 50% i-PrOH).

### Flower color analysis of the petunia transformants

Petunia petals (about 2 g) were frozen for 1 h or longer at −80°C. The pH of the juice obtained by squeezing the petals was measured by a microelectrode (6069-10C, Horiba, Ltd., Kyoto, Japan) on a pH meter (F-22, Horiba, Ltd.). With HPLC, malvidin, which is an anthocyanidin, was identified from the petal extracts and quantified according to a previously reported method [41].

## Results

### The generation of transgenic petunias

**Fig 2:**
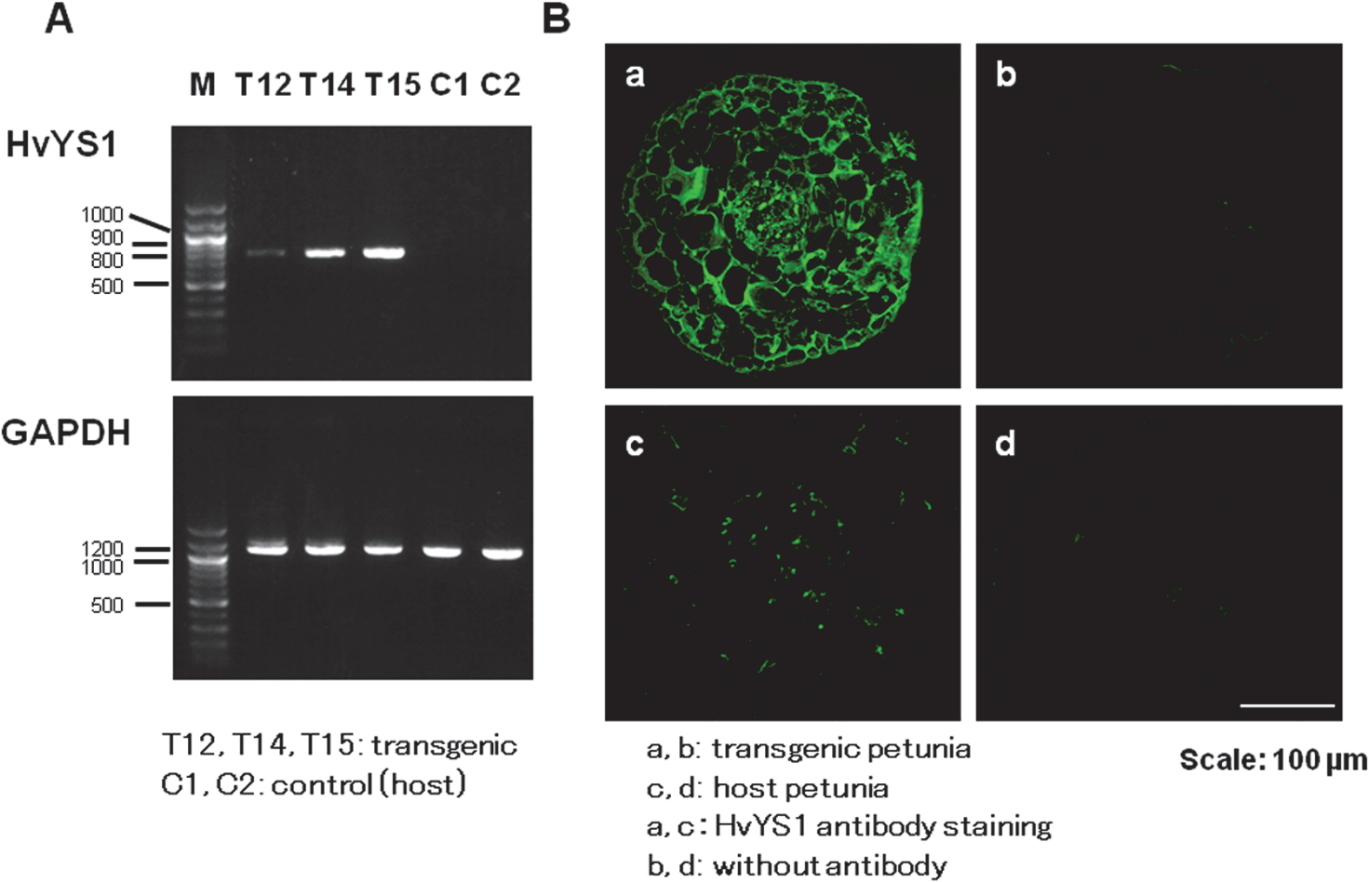
Expression of *HvYS1* in transgenic petunia. (**A**) Agarose gel electrophoresis for the semiquantitative reverse transcription-polymerase chain reaction (RT-PCR) products of *HvYS1* and *GAPDH* in the roots of transgenic petunia (T12, T14, and T15) and host (C1 and C2) plants. (**B**) Tissue localization of HvYS1 protein in transgenic petunia T15 roots (**a**, **b**) and nontransgenic petunia (**c**, **d**) upon treatment with (**a, c**) or without the HvYS1 antibodies (**b**, **d**) [23]. Scale bar: 100 μm.

We generated the transgenic *Petunia hybrid*, which originally had only by Strategy I, by introducing the *HvYS1* MAs-iron complex transporter gene from barley which belongs to Strategy II for iron uptake (Fig. 1). In the 22 transgenic plants, PCR bands corresponding to the *HvYS1* gene were detected at 755 bp in T6, T15 and T16 lines strongly, T13, T14, T20 and T21 lines moderately, and T1, T3, T4, T10, T12 and T22 lines weakly in supporting information (S2 Fig.). Because their amounts were variable, we selected T12 (weakly), T14 (moderately) and T15 (strongly) lines for growth assay (Fig. 2A). In the host petunias (C1 and C2 in Fig. 2A and S2 Fig.; control), the PCR product for the *HvYS1* gene was not detectable, while the PCR product of GAPDH was a similar to the transgenic plants. The localization of HvYS1 protein in the transgenic petunias was examined by immunohistochemistry with a rabbit anti-HvYS1 antibody. The protein was clearly present in the roots of the transgenic plant (Fig. 2B-a), while its expression was hardly detected in the host plant (Fig. 2B-c/d) or the transgenic plant without the antibody (Fig. 2B-b). Thus, transformation of the *HvYS1* gene was successful.

**Fig 3:**
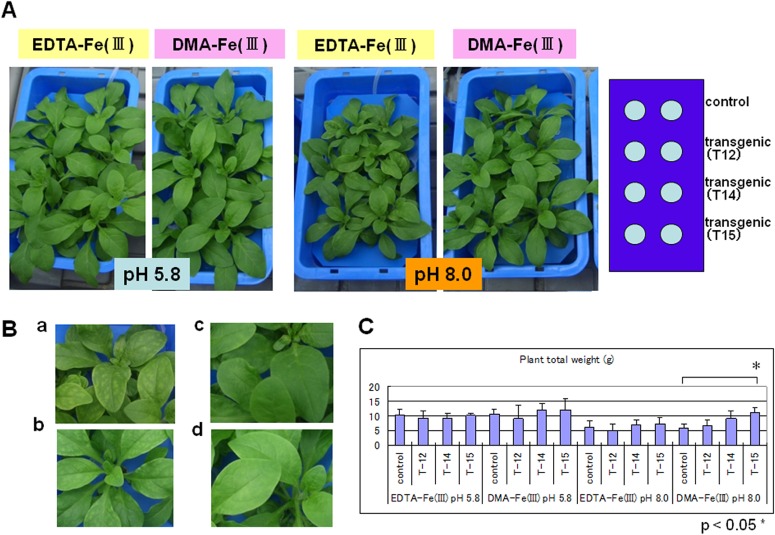
Growth of the transgenic petunias. (A) Growth of the transgenic (T12, T14, and T15 lines) and nontransgenic (control) petunias in hydroponic cultures in the MGRL media containing either synthetic Fe(III)-2′-deoxymugineic acid (DMA) (32) or Fe(III)-ethylenediaminetetraacetic acid (EDTA) (Wako, Japan) at pH 5.8 or pH 8.0. A representative result of two biological replicates is shown. (**B)** Enlarged photos of petunia leaves in panel A with Fe(III)-DMA at pH 8.0; **a**, control; **b**, T12; **c**, T14; and **d**, T15 line. (**C**) The total weight of the plants after a 15-day culture of the plants grown under the conditions in panel A. Asterisks indicate a statistically significant different between T15 line and control petunias (*p < 0.05). n = 3 or 4.

To investigate the alkali tolerance of the petunia transformants (transgenic T12, T14, and T15), the plants were grown in the Fe(III)-DMA/EDTA-containing media at a pH of 8.0 and in the same media at a pH of 5.8. The hydroponic culture, as shown in Fig. 3, was performed by floating the plants on a polypropylene plate over aqueous media. At a pH of 5.8, the petunias, including the host, grew fairly well in the media containing both EDTA and Fe(III)-DMA (Fig. 3A and C), while a clear difference in growth was observed at a pH of 8.0. The T14 and T15 transgenic lines, which exhibited higher levels of the *HvYS1* RNA, showed significantly greater growth than the control host in the media containing Fe(III)-DMA (Fig. 3A and C). The plant did not grow well in alkaline conditions without EDTA or DMA, suggesting that Fe(II) was produced easily by the reduction of Fe(III) in the presence of the chelator. It could then be taken up by Strategy I, which the petunia naturally possesses. The DMA in the present study is a PS of corn and rice, and we have established a highly efficient synthetic route for supplying a sufficient amount of DMA [32]. The alkaline medium that was supplemented with Fe(III)-EDTA caused chlorosis in both the transgenic petunia and the control [Fig. 3A; Fe(III)-EDTA, pH 8.0]. However, the T14 and T15 transgenic lines that were raised in the Fe(III)-DMA-supplemented media grew well [Fig. 3A; Fe(III)-DMA, pH 8.0] and did not show any chlorosis (Fig. 3B-c/d).

**Fig 4:**
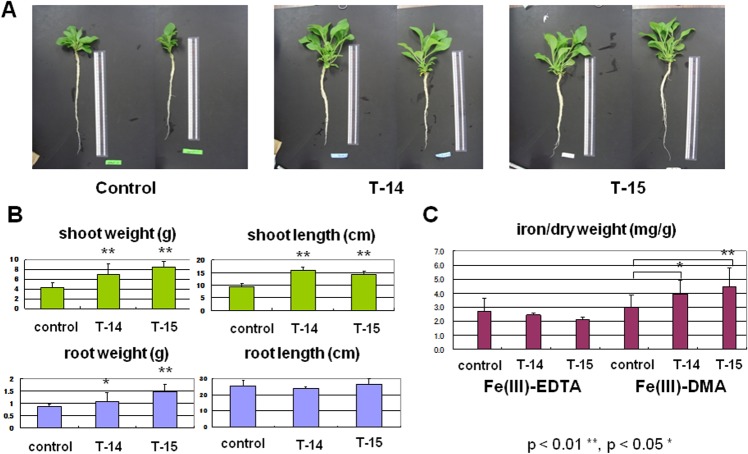
Analysis of transgenic petunia plants in alkaline culture. Shoot and root growth and the iron contents of the transgenic (T14 and T15) and nontransgenic (control) petunias in Fig. 3**A** grown in the alkaline medium (pH 8.0) containing Fe(III)-DMA for 15 days. (**A**) The whole plants of those shown in Fig. 3. A representative result of two biological replicates is shown. (**B**) Shoot and root growth of the transgenic petunias T14 and T15 and the control in weight and length. (**C**) The iron contents of the whole plants of the transgenic petunias grown. Asterisks indicate a statistically significant different between T14, T15 lines and control petunias (*p < 0.05, **p <0.01).n = 3 or 4.

It should be noted that, in the alkaline media containing Fe(III)-DMA, the T14 and T15 transgenic petunia lines showed significantly better growth (Fig. 4A); the shoot weights of the transgenic plants increased 1.5–2.0-fold, and their lengths were 1.5 times longer than that of the host controls (Fig. 4B). The root lengths were not significantly different between the transformants and the hosts, but the weights of the transgenic plant increased 1.2 to 2 fold (Fig. 4B). The iron contents of the transgenic petunia also increased significantly in the presence of Fe(III)-DMA, whereas no particular difference was observed in the Fe(III)-EDTA media (Fig. 4C). These results suggested that the transformed *HvYS1* functions to take iron up in the Fe(III)-DMA form and endows alkaline tolerance to the plant.

### ESI-FT-ICR MS analysis to detect iron and DMA in the petunia roots

**Fig 5:**
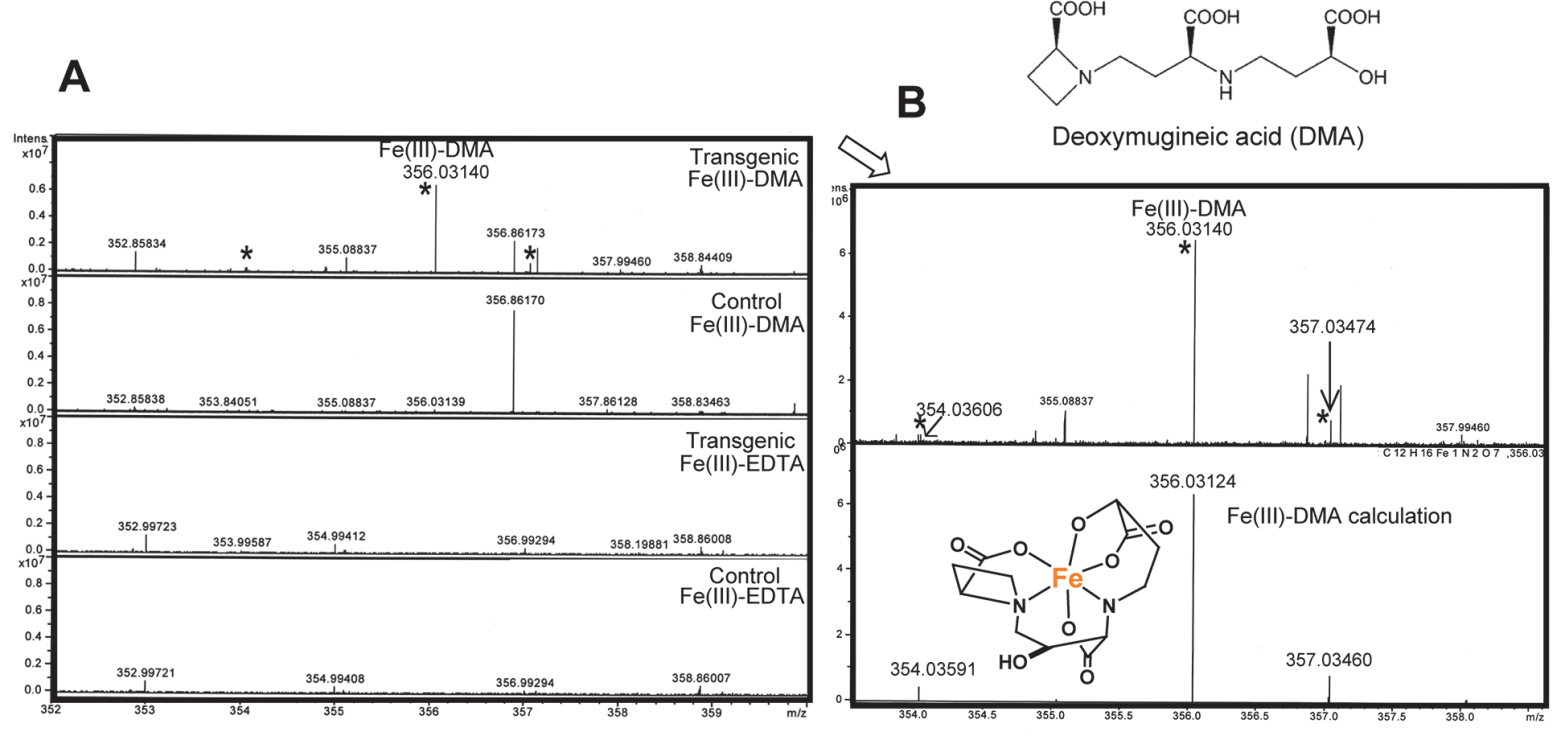
Fourier transform-ion cyclotron resonance mass spectrometry (FT-ICR MS) analysis of transgenic petunia. Detection of Fe(III)-DMA by FT-ICR MS spectra from the root extracts of petunias. (**A**) Molecular peak ([M-4H+^56^Fe(III)]^−^ *m/z* 356.03140) corresponding to the Fe(III)-DMA complex was detected only from the transgenic petunia with *HvYS1* by negative electrospray ionization (ESI)-FT-ICR MS. (**B**) The isotopic ion peaks of Fe(III)-DMA from the transgenic plant root are shown on the top in comparison with the theoretical isotope intensity on the bottom (*). Note that the difference between the observed (*m/z* 356.03140 by detection of nano-ESI(-)-MS) and calculated (*m/z* 356.03124 from the molecular formula of Fe(III)-DMA complex, C_12_H_16_N_2_O_7_Fe_1_: [M-4H+^56^Fe(III)]^−^ by computer software (Data Analysis of Brucker)) values is only +0.45 ppm.

As was implied by the iron contents determined by atomic absorption spectrophotometry (Fig. 4C), the transgenic lines were thought to acquire iron from the hydroponic media in a Fe(III)-DMA complex with the transporter HvYS1. To confirm this, the extracts from their roots were subjected to negative ESI-FT-ICR MS in order to directly detect the Fe(III)-DMA complex. Before the analysis of the transgenic petunia roots, we established the analytical conditions for the highly sensitive detection of MA, which is the PS of barley, and MA in the form of a Fe(III) complex. A simple pretreatment of the crude extracts from barley with a Sephadex G-10 column turned out to be enough to detect the Fe(III)-MA complex with ESI-FT-ICR MS. The fraction corresponding to synthetic Fe(III)-DMA was collected (S3 Fig. and S4 Fig.) and subjected to the MS measurements, which resulted in the appearance of numerous ion peaks (S5 Fig.). Nevertheless, the extremely high resolution of the MS instrument allowed us to identify the molecular ion peak for MA at *m/z* 319.11480 (calc. 319.11469, +1.1 m mass) and its isotopic peaks at *m/z* 320.11817 (calc. 320.11804, +1.3 m mass) and *m/z* 321.11899 (calc. 321.11893, +0.6 m mass), as shown in supporting information (S6 Fig.). The Fe(III)-MA complex was detected *m/z* 372.02632 (calc. 372.02616, +1.6 m mass) with its isotopic ions at *m/z* 370.03097 (calc. 370.03083, +1.4 m mass) and at *m/z* 373.02970 (calc. 373.02951, +1.9 m mass; see S7 Fig.). From a similarly pretreated extract of the root of the transgenic lines that was cultured in the presence of Fe(III)-DMA at pH 5.8, a molecular ion peak corresponding to Fe(III)-DMA complex [M-4H+^56^Fe(III)]^−^ was clearly detected at *m/z* 356.03140 (Fig. 5A) by ESI-FT-ICR MS. In addition, the MS data showed that no peaks corresponding to DMA or Fe(III)-DMA were observed for the roots of the nontransgenic plants in the Fe(III)-DMA-containing medium, thus ruling out the possibility that residual Fe(III)-DMA that was attached to the surface of roots was detected in the transgenic petunia roots. Note that the observed mass of the Fe(III)-DMA ion versus the calculated value at *m/z* 356.03124 differed by only about 0.0016 Da (Fig. 5B). The molecular ion peaks of uncomplexed DMA ([M-H]^−^ at *m/z* 303.11986 calc. 303.11977) was detected only from the *HvYS1*-transgenic petunias (S8 Fig.). These results collectively indicated that the *HvYS1*-transgenic petunia acquired the Fe(III)-DMA complex from the medium through the roots.

### Changes in the flower color of the transformants

**Fig 6:**
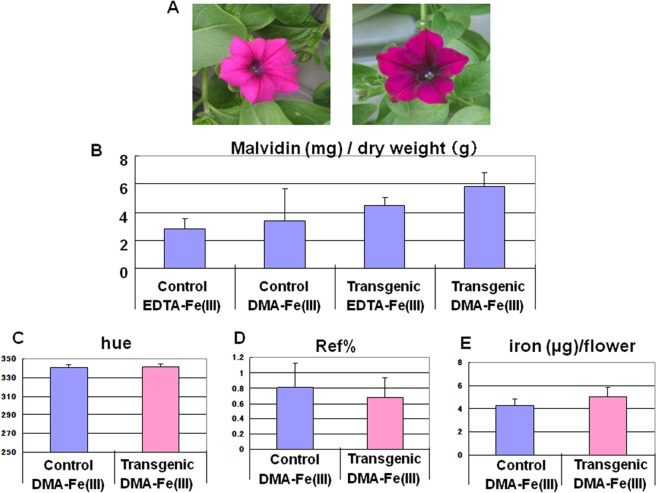
Color change caused by *HvYS1* transformation. (**A**) The comparison with the transgenic petunia flower (T15) (right) and a host (left). Both of the plants were grown in the presence of Fe(III)-DMA at pH 5.8. (**B**) The malvidin contents of the petunias (control; Fe(III)-EDTA n = 3, Fe(III)-DMA n = 3, transgenic; Fe(III)-EDTA n = 4, Fe(III)-DMA n = 8) as indicated in the graph. (**C**) and (**D**) The hue and reflectance in a color-difference meter used to evaluate the color tone and brightness, respectively, of the control (n = 5) and transgenic (n = 12) flowers. (**E**) The iron contents of the whole flower of the control (n = 6) and transgenic (n = 9) petunias grown at pH 5.8.

Because the petunia is a garden plant, we were interested in the transgenic plant’s flower color, which was evaluated by a color difference meter as previously described [41]. In Fig. 6A, the left photograph is of the control plant after 28 days in a hydroponic culture, while the right photograph is of the T15 transgenic line. Clearly, the transformant had darker colored flowers than the control. However, no difference was observed between the hues of the flowers (Fig. 6C). The reflectance (Ref %) of the transgenic flowers was shifted toward a lower value. Therefore, the flower color was darker compared to the host (Fig. 6D). These transgenic flowers contained slightly higher concentrations of iron and malvidin, which is the major flower pigment of this species (Fig. 6B and E), but the pH values of their flower petals were not significantly different with a range of 5.17 to 5.26 compared to the host. The amounts of malvidin were determined for the nontransgenic and transgenic plants in the presence of Fe(III)-EDTA or Fe(III)-DMA (Fig. 6B). These results indicated that the concentration of malvidin in the transformant was approximately two-fold greater than the control with Fe(III)-EDTA; no significant difference was found between the transformant and the control in the presence of Fe(III)-DMA due to the high value of the standard error of the mean in the control group.

## Discussion

### Growth of transgenic petunia

Similar to the mechanisms for barley shown in Fig. 1, rice takes up iron from the soil as a Fe(III)-DMA complex with its original OsYSL15 transporter [27]. Rice also utilizes the OsIRT1 transporter to absorb Fe^2+^, which is relatively abundant under submerged and anaerobic conditions [42]. However, iron is largely present as the insoluble form of Fe^3+^. Thus, extensive studies in genetic engineering have been conducted to enhance the iron absorption of transgenic rice plants with PS-related genes, and these plants have been reported to acquire an alkaline tolerance [43]. The level of tolerance of the barley to iron deficiency is higher than rice because the amount of the PS secretion of barley is more than that in rice [44, 45]. In addition, transgenic rice with PS synthesis [43, 46] and the *HvYS1* transporter genes [47] from barley acquire alkaline tolerance. Yet, there have been no reports on transgenic plants with an iron-PS transport from non-cereal species that lack Strategy II. In this study, therefore, we were interested in the production of a transgenic petunia, which originally has the ability to use only Strategy I, to examine whether the plant became alkaline tolerant by acquiring Strategy II.

Among the 22 lines (T1–T22) of the transgenic petunias with *HvYS1*, three of the strains, T12, T14, and T15, were selected and confirmed to be transformed with *HvYS1* by PCR (S2 Fig.). Among these, the expression levels of T14 was moderately and T15 was strongly, while that of T12 was weakly (Fig. 2A). For this reason, Fe(III)-DMA or Fe(III)-EDTA in the hydroponic media elicited the growth of the three transgenic strains and the control (Fig. 3), despite the fact that their growth was variable. The T12 transgenic plant did not show enhanced tolerance compared to the T14 and T15 lines under the alkaline conditions. At a pH of 8.0 without iron-PS, the petunias failed to survive, indicating that Fe(III)-DMA or Fe(III)-EDTA enabled the original plant to take up iron. As shown in Fig. 1, *Petunia hybrida* can naturally reduce Fe(III) to Fe(II) by ferric chelate reductase, thus enabling the plant to take up iron through the IRT. It is possible that, once Fe(III) is dissolved in the media by DMA or EDTA, the petunias can acquire iron with Strategy I. Thus, the contribution of the newly introduced Strategy II should be evaluated as the difference between the transformant and the control in each graph of Figs. 3 and 4. In the alkaline media containing Fe(III)-DMA, the growth of the T14 and T15 lines was significantly higher than that of the control group (Fig. 3). Similarly, the whole weights and iron contents were significantly increased in the T14 and T15 lines, and their shoot lengths were clearly different (Fig. 4). Furthermore, chlorosis of the leaves was observed in the nontransgenic plants, suggesting that Strategy I alone may not be sufficient to acquire a comfortable amount of iron for the healthy growth of petunias in alkaline conditions. Fe(III)-DMA has been reported to be very useful for plant growth in iron-deficient soil. For example, peanut intercropping with maize in calcareous soil is an effective agroecosystem in which the Fe(III)-DMA that is solubilized by DMA and secreted from maize is acquired directly by neighboring peanuts [48].

### FT-ICR MS analysis of iron-PS complex

There are several reports that iron-PS complex were detected by instrumental methods; e.g., zwitterionic hydrophilic interaction liquid chromatography (ZIC-HILIC) coupled to electrospray ionization mass spectrometry (ESI-MS) [49] and anion exchange liquid chromatography (AE LC) in combination with inductively coupled plasma-mass spectrometry (ICP-MS) [50]. Our preparation method of samples for MS measurement of Fe(III)-DMA from a culture medium could provide a feasible way based on a simple gel filtration, because FT-ICR MS gives rise to ultra-high resolution spectra and very accurate mass data of complex. FT-ICR MS has previously been utilized for detecting synthetic MA iron complexes [51] and cadmium complex [52]. In this study, we detected the molecular ion peak of the Fe(III)-MA complex with FT-ICR MS from the crude extracts of *HvYS1*-transgenic plants after a simple pretreatment with gel permeation chromatography (Fig. 5). The present results demonstrated that the MS technique could be utilized to detect PS-metal complexes with high sensitivity, as shown in Fig. 5A. In addition to the molecular ion peak of Fe(III)-DMA at *m/z* 356.03140 for [M-4H+^56^Fe(III)]^−^ (calc. 356.03124, +1.6 m mass), isotopic ions due to iron were clearly detected at *m/z* 354.03606 for [M-4H+^54^Fe(III)]^−^ (calc. 354.03591, +1.5 m mass) and at *m/z* 357.03474 for [M-4H+^57^Fe(III)]^−^ (calc. 357.03460, +1.4 m mass) (Fig. 5B), unambiguously indicating the presence of an iron atom. Furthermore, iron-free PSs were detected with high accuracy based on their masses [M-H]^−^ at *m/z* 303.11986 (calc. 303.11977, +0.9 m mass) (S8 Fig.).

### Feasibility of *HvYS1* transgenic petunia

As demonstrated by the present results as well as by previous studies [53, 54], the transgenic plants can grow in alkaline soil that is otherwise infertile. Plant physiologists have intensively investigated these issues in order to produce alkaline-tolerant graminaceous plants by transforming enzymes for synthesizing PSs and the transporters of iron-PS complexes, and some of these studies have led to outstanding achievements [55]. The present study may expand these findings toward species outside the graminaceous family; the transgenic petunia with *HvYS1* may open up the breeding of a variety of alkaline-tolerant species. Because barley is known to be one of the most alkaline-resistant plants, the *HvYS1* transporter from barley potentially takes up iron from the soil in severe alkaline conditions, possibly assisting otherwise nonviable plants to grow in barren lands. In addition, a color change of the transformants’ flowers was obvious (Fig. 6), suggesting that the contents of malvidin, rather than iron, were more effectively increased, possibly by enhanced iron uptake, thus implying that the biosynthesis of the flower dyes is controlled by cellular iron content because some of the related enzymes are known to be iron-dependent [56, 57]. These results suggest that transgenic plants with PS-iron transporters might provide a new way to develop new species in horticulture industries.

We showed that *HvYS1*-expressing transgenic petunia lines were grown better than non-transgenic controls under the hydroponic conditions (pH 8.0) containing synthetic Fe(III)-DMA complex. Future experiments under more practical conditions, using soil for example, should further provide information on the feasibility of expressing *HvYS1* as an approach to confer strategy I plants to tolerance to alkaline land when combined with the use of synthetic Fe(III)-DMA fertilizer.

## Supporting Information

S1 Fig Vector of the transformed agrobacterium used to introduce the *HvYS1* translation-region gene into petunias. (TIF)

S2 Fig Agarose gel electrophoresis for the semiquantitative reverse transcription-polymerase chain reaction (RT-PCR) products of *HvYS1* and *GAPDH* in the roots of transgenic petunia lines (T1-T22) and hosts (C1 and C2). (TIF)

S3 Fig MS analysis. Chromatogram (blue trace), monitored at 210 nm, of the extract from barley roots for a test run on a gel filtration column packed with Sephadex G-10 with an HPLC system. The retention time of synthetic DMA-Fe(III) was 32.7 min in a red trace.
(TIF)

S4 Fig MS analysis. Chromatogram, monitored at 330 nm, of the extract from barley that was used for the preparation of a sample for MS analysis under the same conditions as those in S3 Fig.; fractions 31–34 were subjected to Fourier transform-ion cyclotron resonance mass spectrometry (FT-ICR) MS analysis.
(TIF)

S5 Fig MS analysis. FT-ICR MS spectrum of the fractions corresponding to Fe(III)-DMA from barley roots. Full-scan FT-ICR MS in the mass range *m/z* 250–1,700 was acquired with a single microscan.
(TIF)

S6 Fig Identification of mugineic acid (MA) from the extracts of barley roots by FT-ICRMS. The molecular ion peak for MA at *m/z* 319.11480 (calc. 319.11469, +1.1 m mass) and its isotopic peaks at *m/z* 320.11817 (calc. 320.11804, +1.3 m mass) and at *m/z* 321.11899 (calc. 321.11893, +0.6 m mass) are shown on the top in blue and their simulated spectra on the bottom in black.
(TIF)

S7 Fig Detection of the Fe(III)-MA complex by FT-ICR MS from the extracts of barley roots. The molecular ion peak for Fe(III)-MA at *m/z* 372.02632 (calc. 372.02616, +1.6 m mass) and its isotopic peaks at *m/z* 370.03097 (calc. 370.03083 +1.4 m mass) and at *m/z* 373.02970 (calc. 373.02951, +1.9 m mass) are shown on the top and the calculated value is on the bottom.
(TIF)

S8 Fig Deoxymugineic acid (DMA) was detected by FT-ICR MS [negative electrospray ionization (ESI)] from the roots of the transgenic petunia T-14 that was grown in the medium containing Fe(III)-DMA at pH 5.8. The molecular ion peaks of uncomplexed DMA; [M-H]^−^ at *m/z* 303.11986 (calc. 303.11977, +0.9 m mass) and its isotopic peaks at *m/z* 304.12320 (calc. 304.12313, +0.7 m mass) are shown on the top, and the calculated value is on the bottom.
(TIF)
