# Supplementary figures and images for: Transgenic Petunia with the Iron(III)-Phytosiderophore Transporter Gene Acquires Tolerance to Iron Deficiency in Alkaline Environments
Source: PLoS One. 2015 Mar 17;10(3):e0120227. doi: 10.1371/journal.pone.0120227 (PMC4363515; doi:10.1371/journal.pone.0120227)

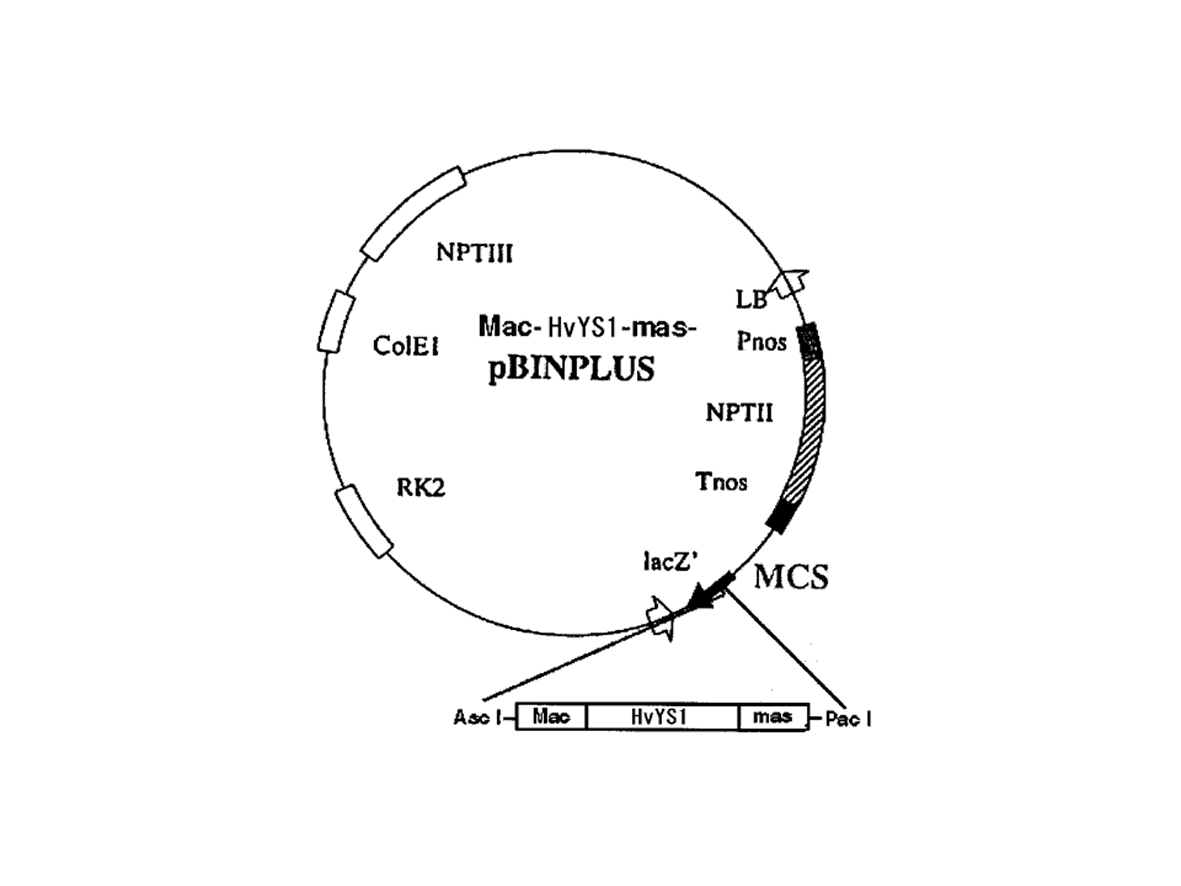

Supplement: S1 Fig — (TIF) [file pone.0120227.s001.tif]

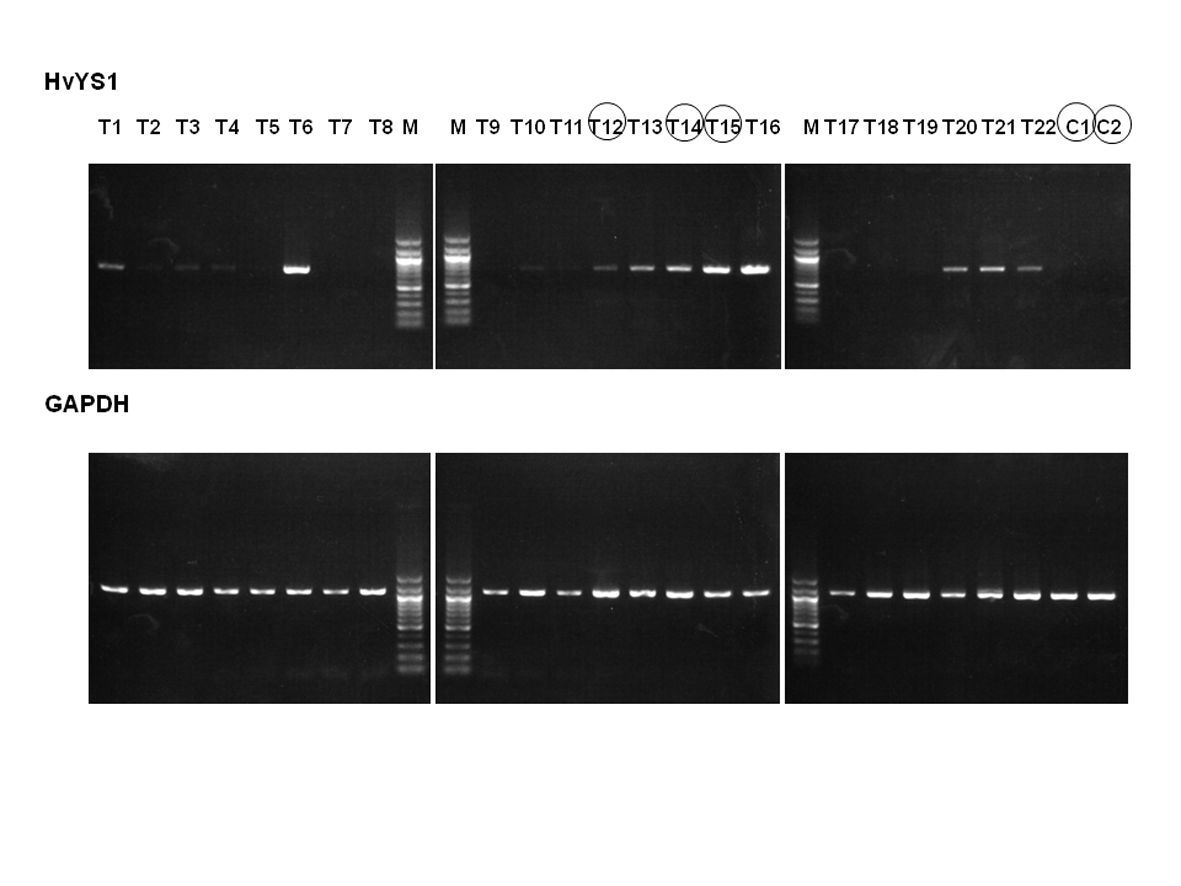

Supplement: S2 Fig — (TIF) [file pone.0120227.s002.tif]

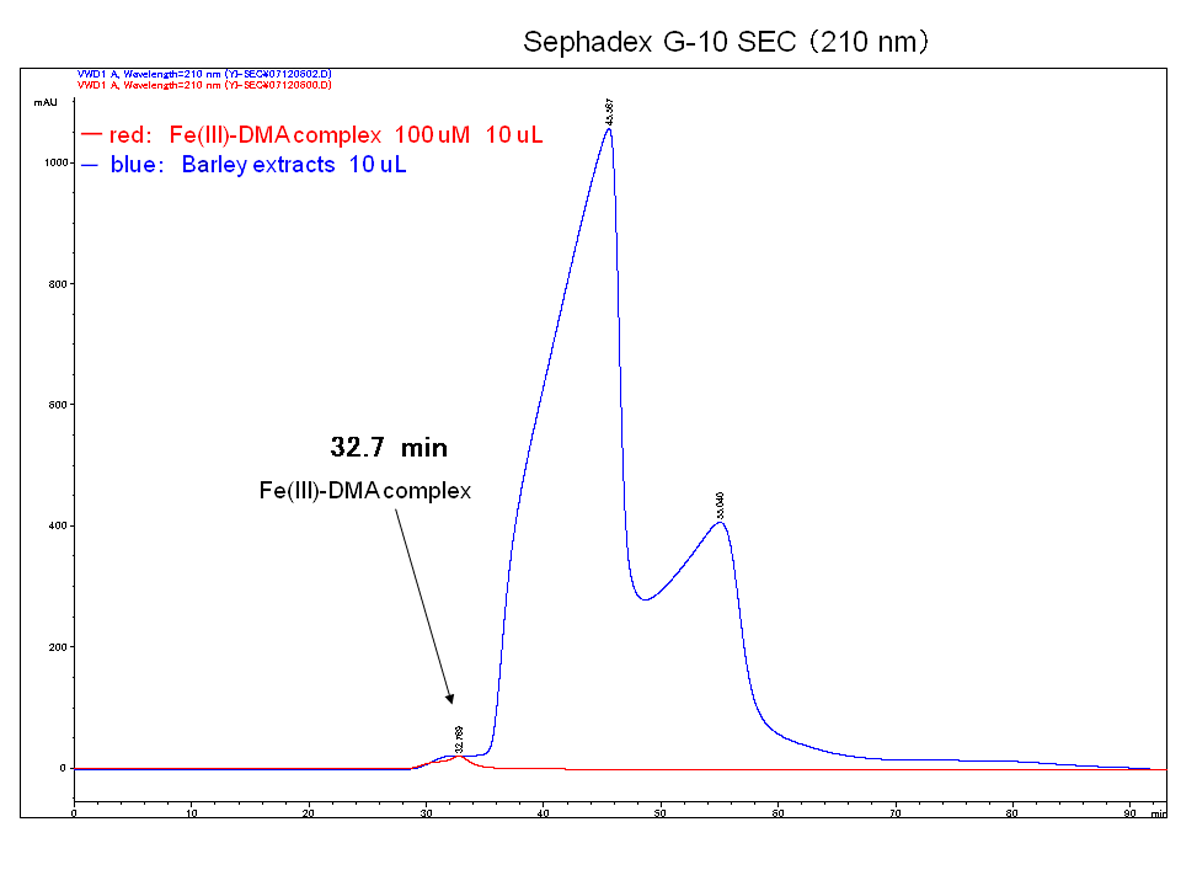

Supplement: S3 Fig — Chromatogram (blue trace), monitored at 210 nm, of the extract from barley roots for a test run on a gel filtration column packed with Sephadex G-10 with an HPLC system. The retention time of synthetic DMA-Fe(III) was 32.7 min in a red trace. (TIF) [file pone.0120227.s003.tif]

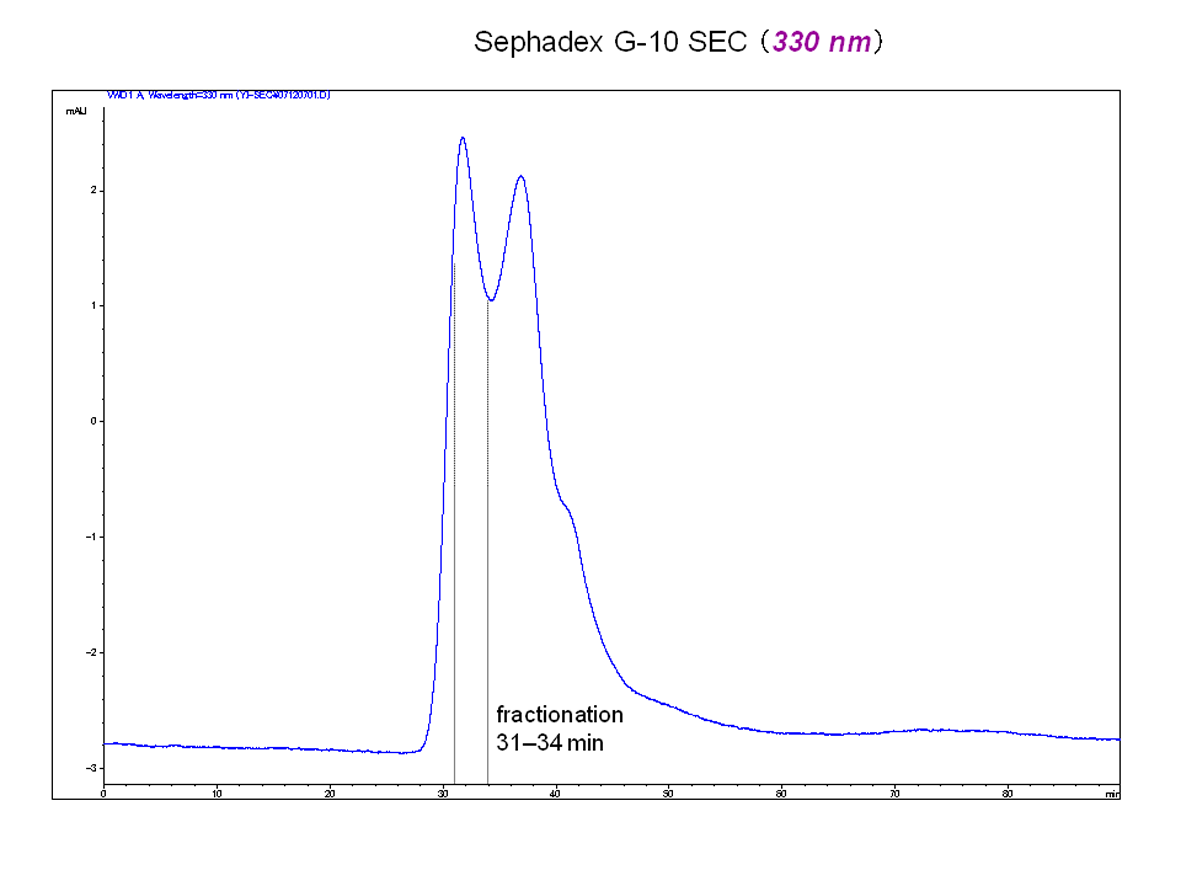

Supplement: S4 Fig — Chromatogram, monitored at 330 nm, of the extract from barley that was used for the preparation of a sample for MS analysis under the same conditions as those in S3 Fig.; fractions 31–34 were subjected to Fourier transform-ion cyclotron resonance mass spectrometry (FT-ICR) MS analysis. (TIF) [file pone.0120227.s004.tif]

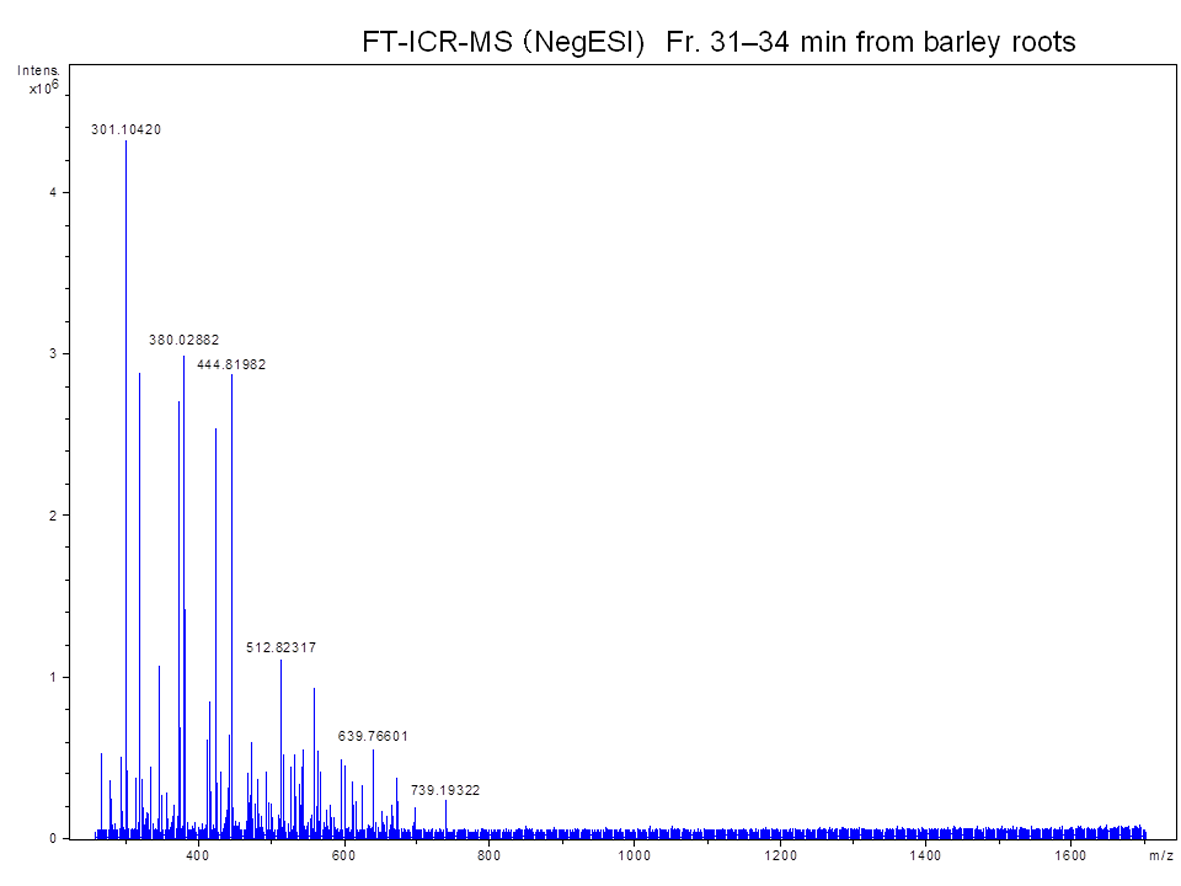

Supplement: S5 Fig — FT-ICR MS spectrum of the fractions corresponding to Fe(III)-DMA from barley roots. Full-scan FT-ICR MS in the mass range m/z 250–1,700 was acquired with a single microscan. (TIF) [file pone.0120227.s005.tif]

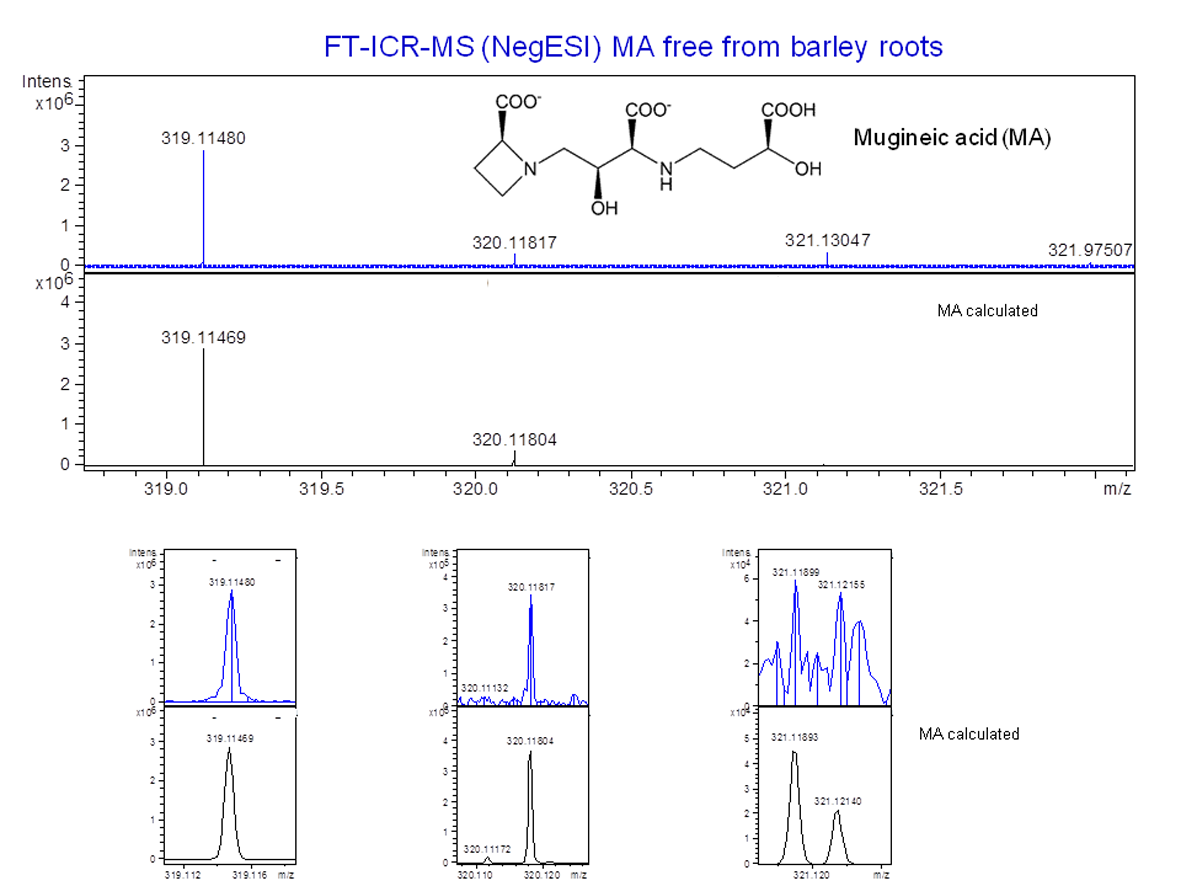

Supplement: S6 Fig — The molecular ion peak for MA at m/z 319.11480 (calc. 319.11469, +1.1 m mass) and its isotopic peaks at m/z 320.11817 (calc. 320.11804, +1.3 m mass) and at m/z 321.11899 (calc. 321.11893, +0.6 m mass) are shown on the top in blue and their simulated spectra on the bottom in black. (TIF) [file pone.0120227.s006.tif]

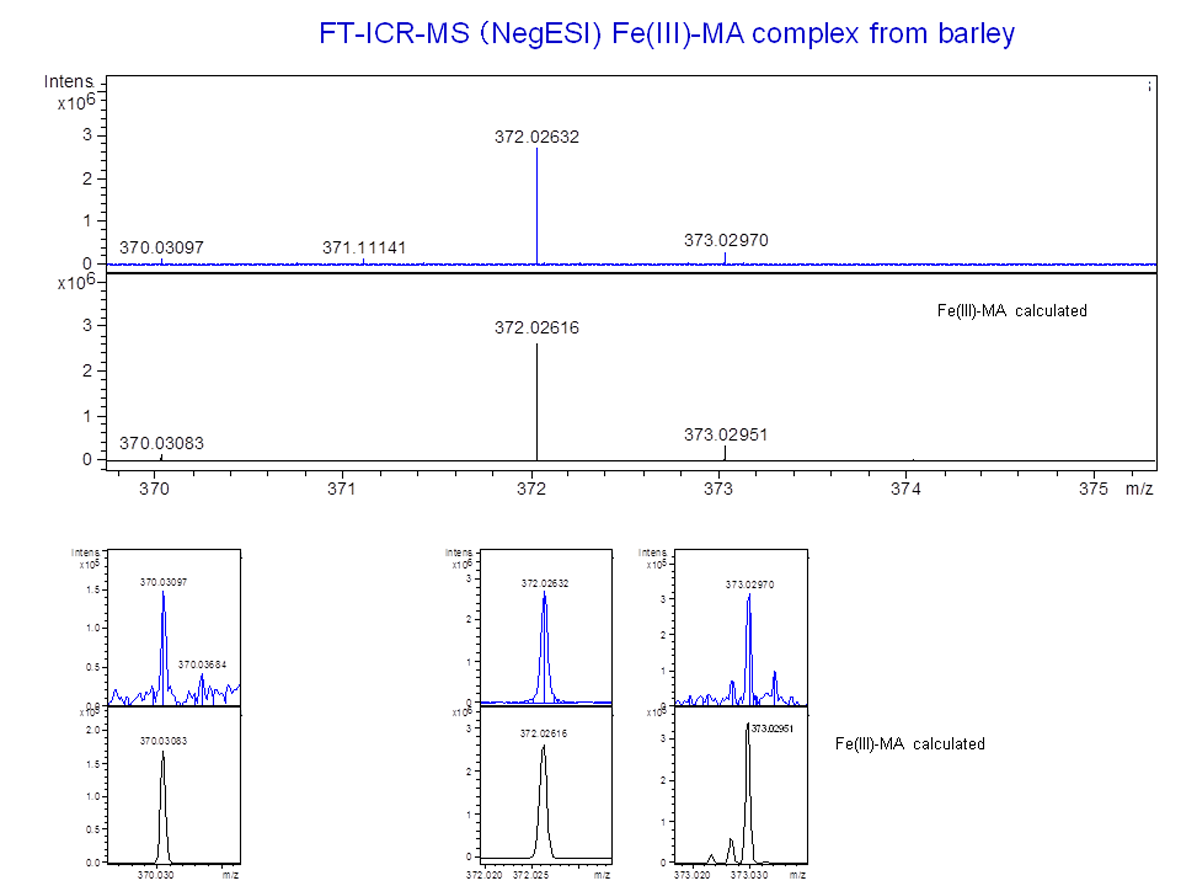

Supplement: S7 Fig — The molecular ion peak for Fe(III)-MA at m/z 372.02632 (calc. 372.02616, +1.6 m mass) and its isotopic peaks at m/z 370.03097 (calc. 370.03083 +1.4 m mass) and at m/z 373.02970 (calc. 373.02951, +1.9 m mass) are shown on the top and the calculated value is on the bottom. (TIF) [file pone.0120227.s007.tif]

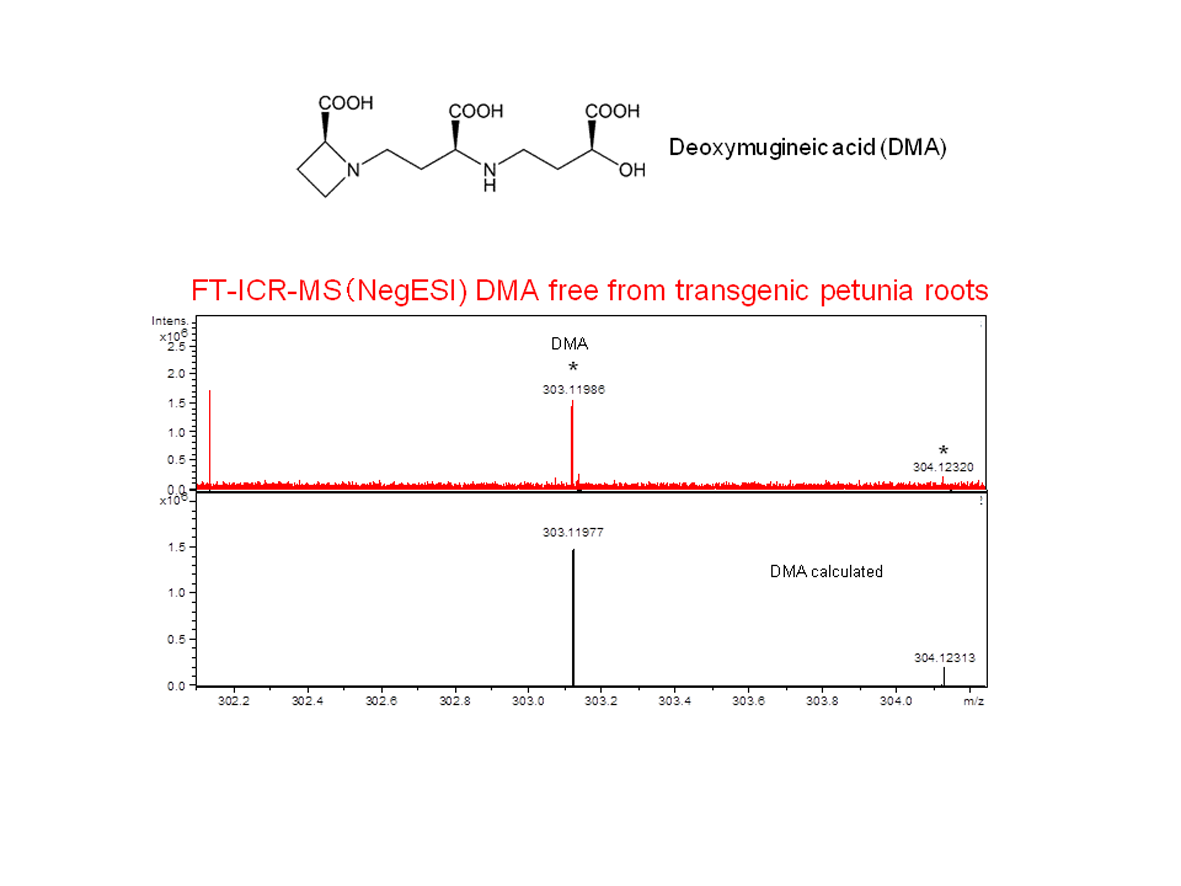

Supplement: S8 Fig — The molecular ion peaks of uncomplexed DMA; [M-H]− at m/z 303.11986 (calc. 303.11977, +0.9 m mass) and its isotopic peaks at m/z 304.12320 (calc. 304.12313, +0.7 m mass) are shown on the top, and the calculated value is on the bottom. (TIF) [file pone.0120227.s008.tif]
